# Tumor repressor gene chondroadherin oppose migration and proliferation in hepatocellular carcinoma and predicts a good survival

**DOI:** 10.18632/oncotarget.19811

**Published:** 2017-08-02

**Authors:** Xiaorong Deng, Weiwei Wei, Niangen Huang, Yumeng Shi, Mingwen Huang, Yehong Yan, Dongjian Li, Jilin Yi, Xinbao Wang

**Affiliations:** ^1^ Department of Gastrointestinal Surgery, The Second Affiliated Hospital of Nanchang University, Nanchang, Jiangxi Province, P.R. China; ^2^ Department of General Surgery, Tongji Hospital, Tongji Medical College, Huazhong University of Science and Technology, Wuhan, Hubei Province, P.R. China; ^3^ Digestive Endoscopy Center, The Second Affiliated Hospital of Nanchang University, Nanchang, Jiangxi Province, P.R. China; ^4^ School of Basic Medical Sciences, Shandong University, Jinan City, Shandong Province, P.R. China; ^5^ Department of Hepatobiliary Surgery, The Second Affiliated Hospital of Nanchang University, Nanchang, Jiangxi Province, P.R. China; ^6^ Department of Hepatobiliary Surgery, The First Affiliated Hospital of Nanchang University, Nanchang, Jiangxi Province, P.R. China; ^7^ Department of Hepatopancreatobiliary Surgery, Zhejiang Cancer Hospital, Hangzhou, P.R. China

**Keywords:** CHAD, prognosis, migration, hepatocellular carcinoma, proliferation

## Abstract

The molecular that used as prognosis and potential therapy target is urgently needed in hepatocellular carcinoma (HCC). In current work, we found the expression of CHAD (chondroadherin) was significantly reduced in hepatocellular carcinoma compared to the normal tissue, on both mRNA and protein levels, in three independent datasets. Survival analysis was implemented on these datasets, and low expression of CHAD was found to be significantly associated with poor survival. Furthermore, metastasis-averse HCC and metastasis-incline HCC group comparison, and protein abundance evaluation of normal-tumor-portal vein tumor thrombus pairs indicate that metastatic tendentiousness is reduced along with CHAD abundance. Correlation analysis was also carried out and CHAD was shown to be significantly associated with differentiation and metastasis. Multivariable cox regression analysis showed that CHAD expression is more important for prognosis, compared to the other clinical indicators. To facilitate the utilization of CHAD clinically, a nomogram was plotted to estimate the three-year survival rate. Functional assays testing the migration and proliferation ability following knock down of CHAD in two cell lines, SMMC7721 and HCCLM3, were performed and discovered that reduction of CHAD level significantly enhance both proliferation and migration in both cell lines. Gene Set Enrichment Analysis (GSEA) comparing the CHAD-low and CHAD-high group showed that KEGG signaling pathways including “focal adhesion”, “ECM receptor interaction”, and “regulation of actin cytoskeleton” were significantly enriched. In conclusion, as a potential prognostic biomarker, tumor suppressor gene CHAD represses migration and proliferation of hepatocellular carcinoma cells, probability via mediating cell-cell adhesion.

## INTRODUCTION

Among the world, hepatocellular carcinoma (HCC) is the fifth leading cancer and the third causes of cancer related deaths [1]. The three-year survival rate of HCC is less than 19% [2] due to the currently limited available drugs and therapy targets. Thus, prognostic biomarker and potential therapy targets are urgently needed to improve the survival of HCC patients and facilitate drug development.

In the past years, efforts have been devoted to screen the molecular biomarkers in predicting survival and offered as potential therapy targets [3–5]. For example, Up-regulated HOXC8 was found to be associated with poor prognosis, and resistance to chemotherapy drugs [6]. Negative regulator of WNT signaling pathway, NKD1, correlated with poor prognosis and enhances the expression of p53 [7]. Overexpression of TCP1, is a negative indicator for survival of HCC [8], and similar trend was observed in another gene SALL4 [9]. MircoRNAs associated with prognosis were also investigated according to recent reports [10–12]. However, the currently used biomarker for prognosis and potential drug is still limited.

In this article, we report that another tumor suppressor gene, CHAD, is significantly lowly expressed in tumor tissues than the normal across three independent datasets. The clinical significance of CHAD, including prognostic effect, clinicopathological indicators association and CHAD expression was evaluated. Functional assays were also carried out to test the migration and proliferation rate following knock down of CHAD. Gene Set Enrichment Analysis (GSEA) comparing high/low CHAD expression group showed that KEGG pathways including “focal adhesion”, “ECM receptor interaction”, and “regulation of actin cytoskeleton” were significantly enriched.

## RESULTS

### CHAD is down-regulated in HCC tissues

**Figure 1:**
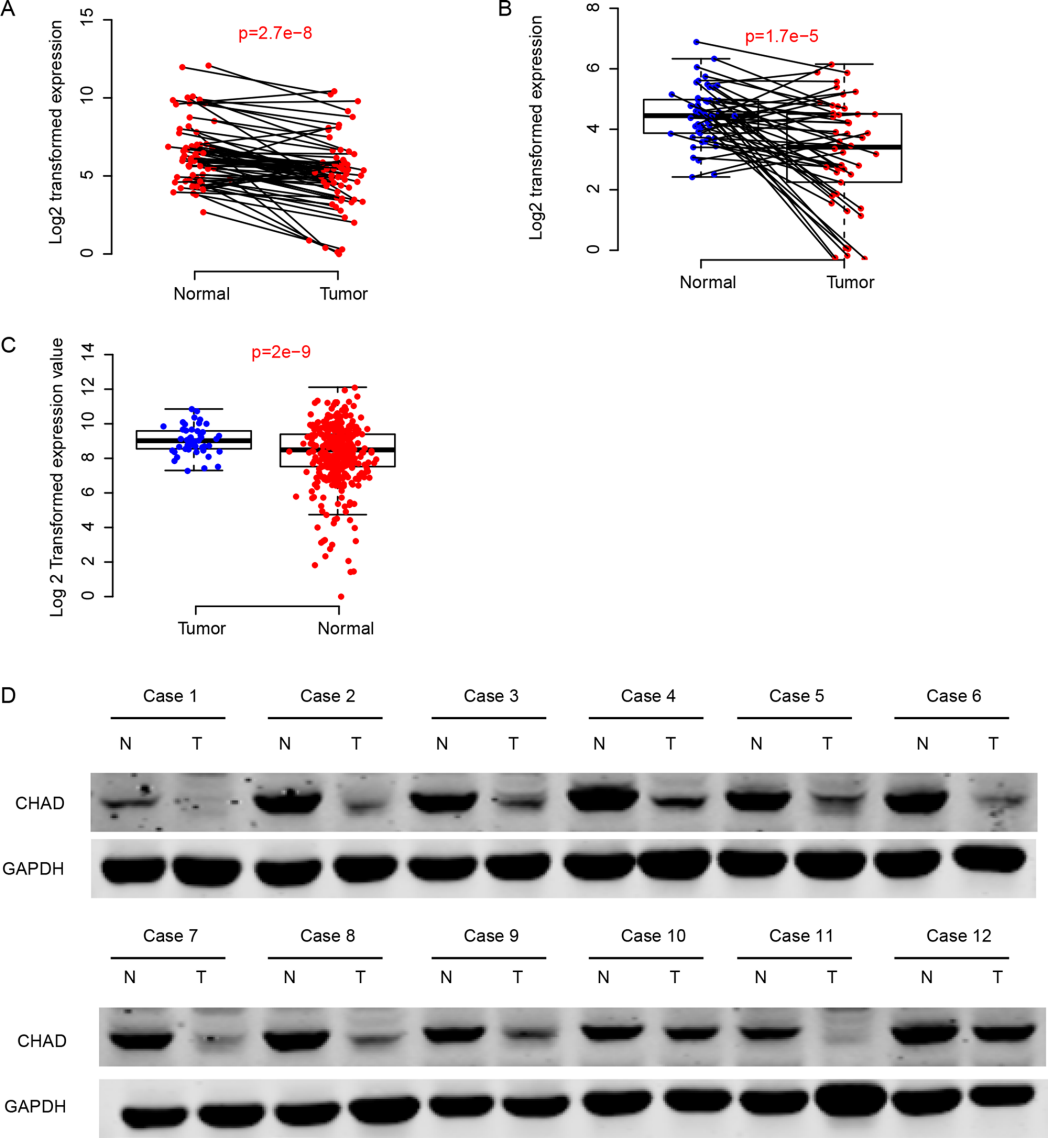
CHAD was down-regulated in hepatocellular carcinoma The mRNA level of CHAD was down-regulated in QPCR (**A**), GEO (**B**), and TCGA-LIHC (**C**) datasets. The protein level of CHAD was also down-regulated compared to the corresponding normal tissues (**D**).

Expression values of CHAD in normal and cancerous tissues were compared in three independent datasets, including QPCR (*N* = 67 pairs), TCGA-LIHC (Normal = 50, Tumor = 269) and GEO dataset (GSE77314, *N* = 50 pairs). The expression of CHAD was significantly enhanced in tumor tissues compared to the (adjacent) normal tissues (Figure 1A–1C). On the hand, the protein abundance in the tumor tissues is also significantly higher than adjacent normal tissues, according to Western Blot results in 30 paired samples (Figure 1D). All these results above indicate that CHAD is down-regulated in HCC tissues.

### CHAD is a prognostic marker for HCC

**Figure 2:**
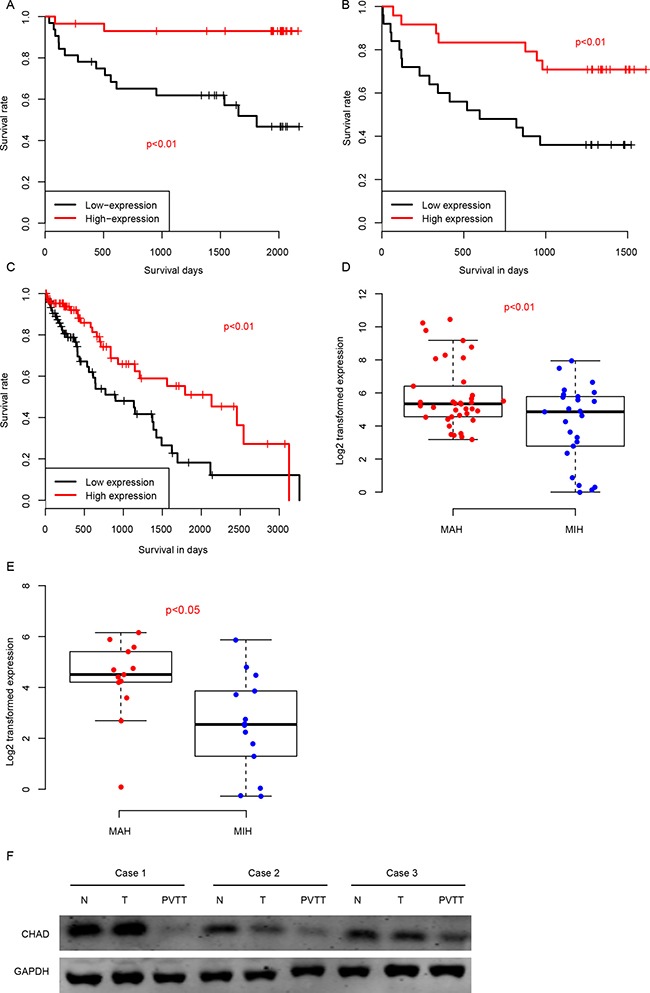
The high expression level of CHAD predicts a good survival in QPCR (**A**), RNA-seq (**B**), and TCGA-LIHC (**C**) datasets. In addition, MAH CHAD expression was significantly higher than MIH group in both QPCR (**D**) and RNA-seq (**E**) group. PVTT CHAD expression was also significantly lower than primary tumor tissues on protein level (**F**).

We also evaluated the prognostic effect of CHAD by dividing the samples into CHAD-high and CHAD-low group according to the median expression value in GSE77314, QPCR and TCGA-LIHC datasets. The CHAD-high group had a significantly longer survival time than CHAD-low group in these three datasets (Figure 2A–2C, respectively). In addition, we classified the samples into metastasis-averse HCC (MAH) and metastasis-incline HCC (MIH) group based on the clinicopathological indicators and follow up information, and compared the relative expression values of CHAD in both QPCR and GSE77314 datasets. As expected, the CHAD expression in MIH group is significantly lower than MAH group (Figure 2D, 2E). Portal vein tumor thrombus (PVTT) are HCC cells migrate from primary tumor site to portal vein, thus the metastatic ability of cells in PVTT is stronger than the resident tumors. We compared the protein abundance in normal-tumor-PVTT pairs, and the result showed that PVTT had a significantly lower level of CHAD than the primary tumor tissues (Figure 2F). In summary, high expression CHAD is associated with less metastasis, and predicts a good survival.

### Clinicopathological indicators and CHAD

The correlation between clinicopathological and CHAD expression was evaluated in qPCR dataset, as shown in Table 1. Among these indicators, we noted that distant metastasis is significantly associated with CHAD, 34.8% (8/23) patients in CHAD-low group detected distant metastasis from the primary organ, while only 4.3% (1/23) of CHAD-high group exhibited metastasis (*p* = 0.022). Besides, differentiation level of these samples was also significantly different. 93.8% (30/32) samples in CHAD-low group was highly differentiated while 70.6% (24/34) in CHAD-high group was identified to be highly differentiated (*p* = 0.0235). The other clinical information including HBV infection, age, gender, AFP (alpha fetoprotein) level, and membrane and recurrence were not significantly correlated.

**Table 1:** Clinical information significantly associated with CHAD

Variables	CHAD-Low	CHAD-high	*p*-value
**Age**			0.784
< 60	25	24	
> 60	8	10	
**HBV infection**			1
No	5	6	
Yes	27	28	
**Differentiation**			**0.0235**
Low (1–2)	2	10	
High (3–4)	30	24	
**Diameter**			0.144
< 5 cm	12	19	
> 5 cm	21	15	
**Membrane**			0.534
No	12	18	
Yes	9	7	
**AFP**			0.127
< 20	9	15	
> 20	24	17	
**Gender**			1
Male	31	31	
Female	2	1	
**Reccurence**			0.768
No	14	17	
Yes	8	8	
**Metastasis**			**0.022**
No	15	22	
Yes	8	1	

**Figure 3:**
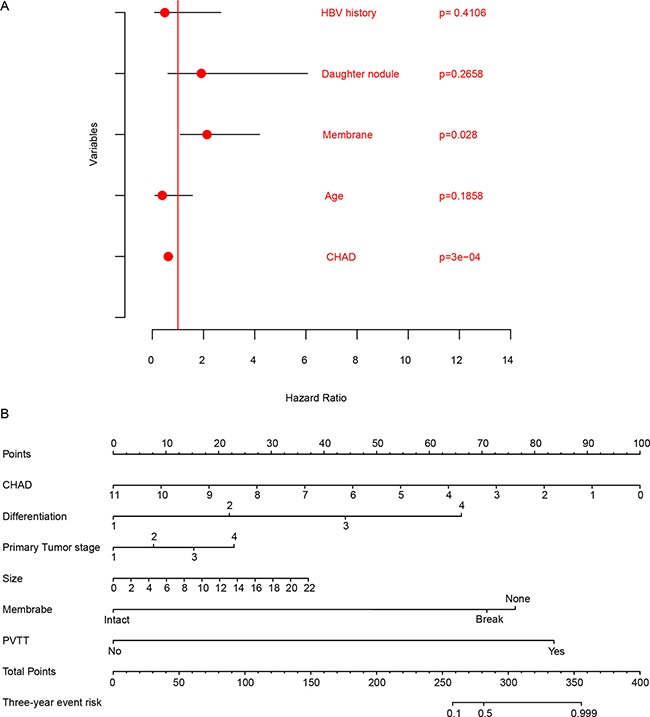
Comparison of clinicopathological information and CHAD in prognosis The multivariate cox regression showed that CHAD was significantly associated with survival (**A**), and nomogram show that CHAD spanned the most range compared to other clinical information (**B**).

To test the significance of CHAD expression and other clinical information, multivariate cox regression was implemented. We noticed that both CHAD expression and membrane existence were significantly associated with survival, while the other information including HBV history, daughter nodule and age were not (Figure 3A). To facilitate the utilization of CHAD clinically and evaluate the importance of CHAD, a nomogram for three-year survival was plotted to integrate clinical information and CHAD expression (Figure 3B). The CHAD contributed the most risk points (0–100) compared to the other clinical information. All these results above indicate that CHAD was an important clinical indicator for prognosis and associated with differentiation and metastasis.

### CHAD opposes proliferation and migration in HCC cell lines

**Figure 4:**
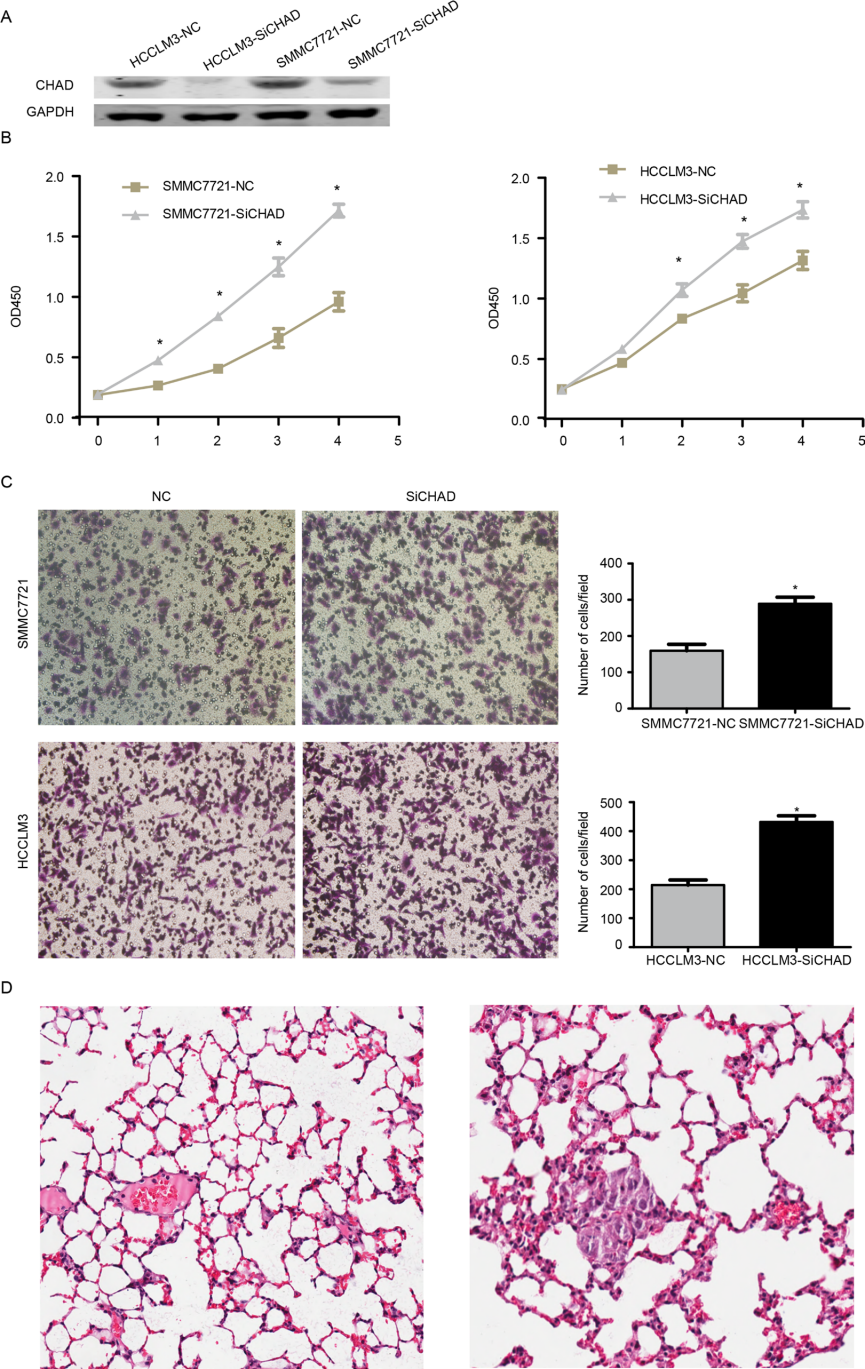
CHAD oppose proliferation and migration of HCC After knock down of CHAD (**A**), the proliferation rate of both SMCC7721 (**B**, left) and HCCLM3 (B, right) was significantly enhanced. The migration rate of SMCC7721 (**C**, top) and HCCLM3 (C, bottom) was significantly increased along with knock down of CHAD. * indicates statistically significant (*p* < 0.05). The non-lung metastatic SMMC7721-CHAD and lung metastatic SMMC7721-GFP (**D**, left and right, respectively).

Clinical information analyses and MAH-MIH group analysis have revealed that CHAD was associated with cell metastasis. For further validation, we assessed both the migration and proliferation rate of two different HCC cell lines, SMMC7721 and HCCLM3, following knock down of CHAD using siRNAs (Figure 4A). Proliferation rate was evaluated using CCK8 kit every 24 hours, and the result show that the proliferation ability of HCC cells was significantly enhanced in CHAD knock down group, in both cell lines (Figure 4B).

The migration assay was also measured following CHAD knock down using the same method. After incubation for 14 hours, the migrated cells were compared (Figure 4C). Migrated cells in the CHAD knock down group were significantly less than the control group, in both SMMC7721 and HCCLM3 cell lines.

After rejecting the SMMC7721 cell line overexpressing CHAD and GFP (termed SMMC7721-CHAD and SMMC-7721-GFP) to naked mouse tail vein (each 5 replicates), the lungs were dissected and stained after 18 weeks to identify the lung metastatic status. Cancer cells were detected in one of the five mouse in SMMC7721-CHAD group, while four out of five were detected in SMMC-GFP group (Figure 4D). All these results indicate that aberrant expression of CHAD is associated with migration and proliferation of HCC cells both *in vivo* and *in vitro*.

### Pathways associated with CHAD expression

**Figure 5:**
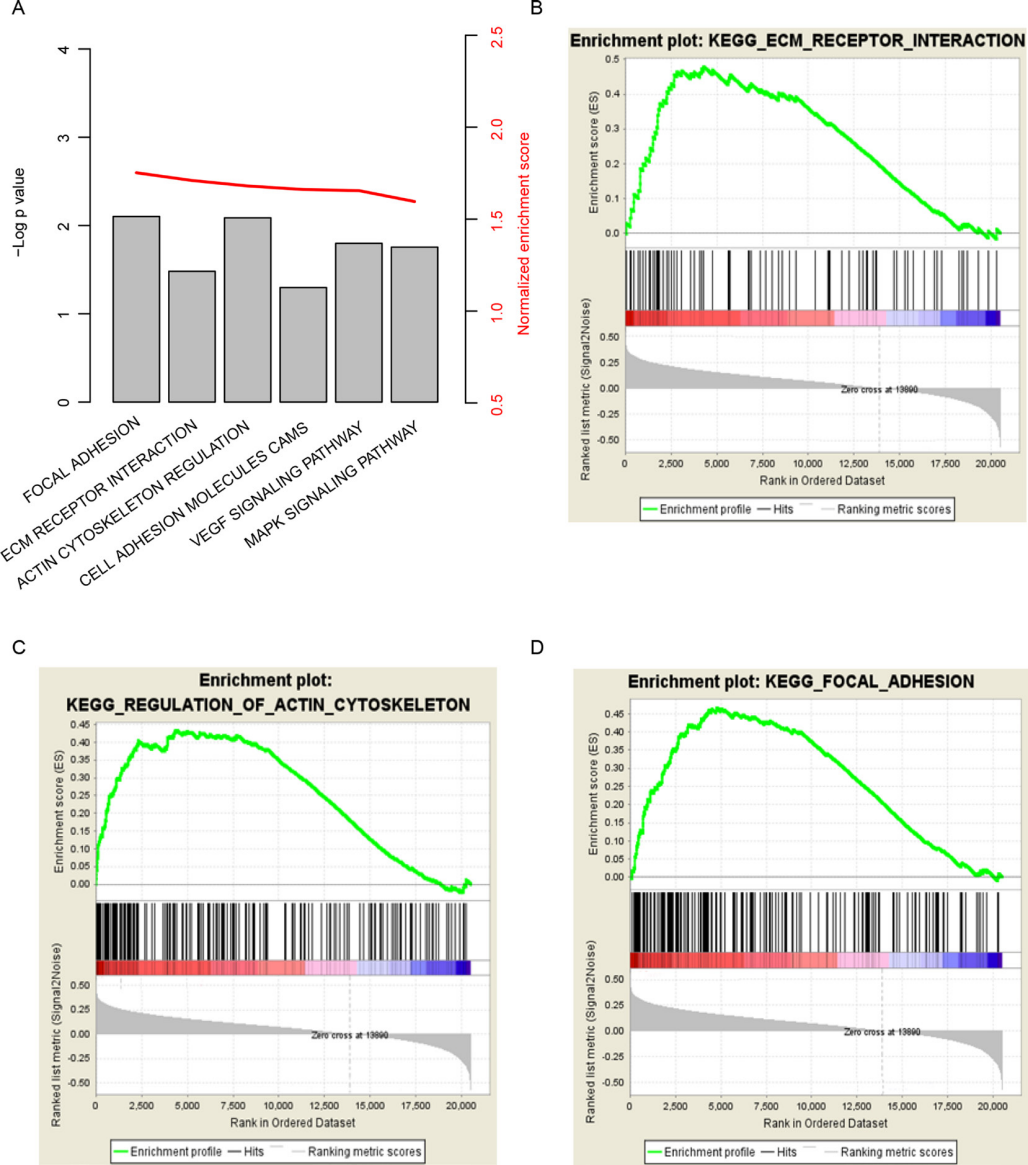
KEGG pathways associated with CHAD expression GSEA results showed that cell-cell interaction related pathways significantly associated with CHAD (**A**), including “focal adhesion” (**B**), “ECM receptor interaction” (**C**), and “regulation of actin cytoskeleton”(**D**).

In order to investigate the pathways that CHAD may regulate, Gene Set Enrichment Analysis was carried out using GSEA java software, by comparing expression of genes in the CHAD-high/low group divided by median expression level of CHAD. KEGG (Kyoto Encyclopedia of Genes and Genomes) signaling pathways was used as reference in this step to evaluate the pathways CHAD may modulate. Carcinogenesis and development associated pathways, including “focal adhesion”, “ECM receptor interaction”, and “regulation of actin cytoskeleton” were identified as significantly altered along with aberrant CHAD expression (Figure 5A). We noted that genes involved in focal adhesion signaling pathway was significantly altered in CHAD-low group, which may explain the high morality and metastasis rate (Figure 5B). In addition, extracellular matrix related pathways were also observed to be associated with CHAD expression (Figure 5C, 5D). In summary, CHAD expression alters prognosis of hepatocellular carcinoma may via altering cell adhesion related signaling pathways.

## DISCUSSION

Lack of reliable potential prognostic biomarkers and therapy target makes HCC prognosis and treatment difficult. In current work, by analyzing the expression of CHAD in HCC, we investigated the prognostic effect of CHAD and studied the function of CHAD on proliferation and migration. The results indicate that CHAD is a potential diagnostic and prognostic biomarker for HCC, and aberrant expression of CHAD significantly alters the proliferation and migration ability of HCC cell lines. Potential pathways associated with CHAD expression includes “focal adhesion”, “ECM receptor interaction”, and “regulation of actin cytoskeleton”. All these results indicate that CHAD is a potential biomarker for HCC prognosis.

CHAD is known as cartilage matrix protein thought to mediate adhesion of isolated chondrocytes [13–15]. However, reports regarding other function of CHAD is still limited, hitherto, including the role of CHAD during carcinogenesis and cancer development. The expression of CHAD was detected in both hepatocellular carcinoma and normal hepatocytes, suggesting that CHAD may also play some roles in liver cells. Since CHAD mediates cell matrix adhesion of chondrocytes. Thus it is suspect that CHAD also involved in cell-cell interaction in hepatocytes and hepatocellular carcinoma. Functional analysis and clinical correlation results indicated that CHAD was down-regulated and associated with poor survival and metastasis, which was consistent with this hypothesis. Focal adhesion and ECM receptor [16, 17] was shown to be associated with liver cancer metastasis. GSEA results showed that KEGG signaling pathways including “focal adhesion”, “ECM receptor interaction”, and “regulation of actin cytoskeleton” was significantly enriched and altered along with CHAD expression, which also supports our hypothesis.

The most important limit of this study is that the detailed mechanism of CHAD modulate cell-cell interaction is still not clear. Another limit is that samples involved in this study were also retrospective samples. Thus the clinical utilization CHAD as clinical biomarker needs more comprehensive study.

In summary, in current work, we identified a novel prognostic biomarker, CHAD, was down-regulated in hepatocellular carcinoma cells, oppose cell proliferation and migration, predicts a good survival of HCC patients, and may serve as a potential therapy target.

## MATERIALS AND METHODS

### QRT-PCR quantifies CHAD

Written informed consent was obtained from all patients in this study and this study is approved by The Second Affiliated Hospital of Nanchang University Ethnic Committee. Isolate total RNA from cancer and normal tissues using Trizol (Invitrogen, CA, USA) following the guiding protocols. Assess the quantity and quality of RNA with Nanodrop 2000 (Thermo Scientific, USA). Using random primers and M-MLV Reverse Transcriptase (Invitrogen, CA, USA) to synthesize the first strand cDNA from 3 μg total RNA. Quantify relative expression of CHAD use real-time polymerase chain reaction (RT-PCR) with an ABI PRISM 7900 sequence detector (Applied Biosystems, Carlsbad, CA, USA) and SYBR Green (Applied TaKaRa, Japan) according to the manufacture provided protocols. Normalize the relative expression values generated from different batches with endogenous control, 18S RNA, and CT values. All samples tested were in duplicate, and mean values were retained for further analysis.

### Cell culture and transfection

The siRNA for CHAD was purchased from Biomics biotechnologies Co. (Shanghai, China). The sense sequence is: UGACCUUGUUGUGGUCCAGdTdT and UUAUUGGUAAGGGCGAGGGdTdT. The siRNA transfection was performed with INTERFERin reagents (Poly plus) according to the manufacturers' instructions. Briefly, for each well (6 well, for example), dilute 11 μl (20 μM storage concentration) siRNA duplexes into 200 μl of medium without serum. Mix by pipetting up and down. Then, add 12 μl of INTERFERin into the 200 μl of siRNA duplexes, homogenize by vortex immediately for 10 seconds. Incubate for 10 minutes at room temperature to allow transfection complexes to form between siRNA duplexes and INTERFERin. Then, add 200 μl of transfection mix into the 2 ml cell culture medium to complete a final concentration of 100 nM siRNA. Finally, homogenize by gently swirling the plate.

### Western blot

Protein was extracted with RIPA Lysis Buffer according to the manufacturer provided protocols, and then centrifuged at 12,000 rpm for 15 minutes. The total protein concentration of samples was evaluated with the standard bicinchoninic acid assay. The CHAD antibody (Abcam, Shanghai, China) was diluted with 1:500 (endogenous control GAPDH at 1:10000, Santa Cruz Biotechnology) and immuno-complexes were further incubated with the fluorescein-conjugated secondary antibody. The antibody binding signal intensity was detected with Odyssey infrared scanner (Li-CorBiosciences, Inc.)

### Migration and proliferation assay

Use trans-well filter chambers to assess the migration ability of HCC cell lines (Costar, Corning, NY, USA) complying protocols provided by manufacturer. Re-suspend ∼1 × 10^5^ cells in serum-free medium, gently shock several times, add these cells into the top of the chamber, and add medium containing 10% FBS into the other side of chamber. Incubate the cells for 12 hours, stain, photograph, and count the cells on the lower surface of the membrane using a microscope in three random fields per field for each assay. All experiments were performed three times. For the cell proliferation assay, HCC cells were seeded into a 96-well plates (3000/well) and use Cell Counting Kit-8 (Dojindo Laboratories, Japan) per 24 hours, following the manufacturer provided protocols to test the proliferation rate.

### *In Vivo* metastatic assay

Teen 6-week-old male nude mice were randomized into two groups (*N* = 5, for each group). SMMC7721-CHAD and SMMC-GFP cells (5 × 10^5^) were injected into the tail vein. Mice were sacrificed at 18 weeks post injection and lungs were dissected and H&E staining for lung metastatic foci detection. Animals were housed in cages under standard conditions, following the requirements of the Second Affiliated Hospital of Nanchang University Animal Care Facility and the National Institutes of Health guidelines.

### Statistical analysis

All data analysis and graphing was performed with R and R packages. The receiving operating characteristic curve was plotted and calculated with R package “pROC” [18]. Survival analyses was implemented with R package “survival”, and Gene Set Enrichment analysis was carried out with GSEA java software [19]. The KEGG curated pathways were downloaded as reference pathways and 1000 permutation were used. Correlation between CHAD expression and clinical information was evaluated with fisher's exact test. The CHAD-high and CHAD-low expression group was defined by the median expression value of CHAD, as cutoff.
